# Prospective cohort study for assessment of integrated care with a triple aim approach: hospital at home as use case

**DOI:** 10.1186/s12913-022-08496-z

**Published:** 2022-09-07

**Authors:** Carme Herranz, Rubèn González-Colom, Erik Baltaxe, Nuria Seijas, Maria Asenjo, Maaike Hoedemakers, David Nicolas, Emmanuel Coloma, Joaquim Fernandez, Emili Vela, Isaac Cano, Maureen Rutten-van Mölken, Josep Roca, Carme Hernandez

**Affiliations:** 1 Consorci d’Atenció Primària de Salut de L’Eixample (CAPSBE), Barcelona, Spain; 2 Institut d’Investigacions Biomèdiques August Pi I Sunyer (IDIBAPS), Barcelona, Spain; 3 Institute of Pulmonary Medicine, Chaim Sheba Medical Center, Tel Hashomer, Ramat Gan, Israel; 4 Hospital Clínic de Barcelona, Villarroel 170, 08036, Barcelona, Spain; 5 Erasmus School of Health Policy and Management and Institute for Medical Technology Assessment, Erasmus University Rotterdam. Rotterdam, The Netherlands, Rotterdam, Netherlands; 6 Àrea de Sistemes d’Informació. Digitalization for the Sustainability of the Healthcare System (DS3), Servei Català de La Salut, Barcelona, Spain

**Keywords:** Chronic care, Health Delivery Assessment, Health Services Research, Hospital at Home, Implementation Science, Multiple Criteria Decision Analysis, Triple Aim

## Abstract

**Background:**

Applicability of comprehensive assessment of integrated care services in real world settings is an unmet need. To this end, a Triple Aim evaluation of Hospital at Home (HaH), as use case, was done. As ancillary aim, we explored use of the approach for monitoring the impact of adoption of integrated care at health system level in Catalonia (Spain).

**Methods:**

Prospective cohort study over one year period, 2017–2018, comparing hospital avoidance (HaH-HA) with conventional hospitalization (UC) using propensity score matching. Participants were after the first episode directly admitted to HaH-HA or the corresponding control group. Triple Aim assessment using multiple criteria decision analysis (MCDA) was done. Moreover, applicability of a Triple Aim approach at health system level was explored using registry data.

**Results:**

HaH-HA depicted lower: i) Emergency Room Department (ER) visits (*p* < .001), ii) Unplanned re-admissions (*p* = .012); and iii) costs (*p* < .001) than UC. The weighted aggregation of the standardized values of each of the eight outcomes, weighted by the opinions of the stakeholder groups considered in the MCDA: i) enjoyment of life; ii) resilience; iii) physical functioning; iv) continuity of care; v) psychological wellbeing; (vi) social relationships & participation; (vii) person-centeredness; and (viii) costs, indicated better performance of HaH-HA than UC (*p* < .05). Actionable factors for Triple Aim assessment of the health system with a population-health approach were identified.

**Conclusions:**

We confirmed health value generation of HaH-HA. The study identified actionable factors to enhance applicability of Triple Aim assessment at health system level for monitoring the impact of adoption of integrated care.

**Registration:**

ClinicalTrials.gov (26/04/2017; NCT03130283).

**Supplementary Information:**

The online version contains supplementary material available at 10.1186/s12913-022-08496-z.

## Background

Over the last decade, implementation science [1, 2] has experienced significant progresses resulting in well-accepted conceptual frameworks for assessment of healthcare services [3–6]; that should cover the following three areas: i) deployment strategies aiming at identifying barriers/facilitators for service adoption; ii) comprehensive approaches to assess outcomes; and iii) identification of key performance indicators (KPI) feeding customized dashboards for continuous long-term quality monitoring of complex interventions [7–10]. Applicability of such recommendations for evaluation of integrated care services (ICS) [3, 11–13] in real-world settings is still an issue limiting service adoption, as well as comparability and transferability across sites [14, 15].

With respect to the comprehensive assessment of outcomes, the Triple and Quadruple Aim approaches [16, 17] are consolidated conceptual strategies for evaluation of value generation [18] of ICS. The Triple Aim takes into consideration the following three outcome categories: i) health and well-being; ii) patients’ experience and perception of care; and iii) costs; whereas the Quadruple Aim approach incorporates healthcare professionals’ engagement as an additional key determinant to assess healthcare delivery.

The current research should be envisaged as part of the strategy for exploring applicability of a comprehensive framework for evaluation of ICS, covering both vertical and horizontal integration, in the region of Catalonia (Spain) [19]. To this end, we analyzed feasibility of the Triple Aim approach at health system level aiming at monitoring the impact of the process of large-scale adoption of integrated care in Catalonia during the period 2011–2017. Accordingly, a retrospective population-health study was done using registry data [20, 21].

However, the main objective of the current study was to prospectively assess value-based healthcare of Hospital at Home (HaH) over one-year period (2017–2018), as an example of ICS. To this end, information obtained from a Triple Aim assessment of HaH was elaborated using a Multiple Criteria Decision Analysis (MCDA) [22–24], as an academically sound methodology for evaluation of health value generation, aiming to provide the basis for innovative reimbursement incentives and financial sustainability of health-services.

## Material and methods

### The context

HaH [25–27] refers to home-based delivery of acute hospital services to patients for a condition that otherwise would require acute hospital inpatient care. Such a modality of care encompasses home-based, short-term, services aiming at fully or partially substituting conventional hospitalization; that is, Hospital Avoidance (HaH-HA) and Early Discharge (HaH-ED), respectively.

In 2006, Hospital Clínic de Barcelona, a tertiary reference centre covering a population of 520 k citizens pioneered the deployment of HaH as a mainstream transversal service [28–30]. A recent Cost-Consequence Analysis (CCA) of HaH-HA (Carme H, Carme H, Erik B, Nuria S, Ruben G, Asenjo M, David N, Enric C, Fernandez J, Isaac C, Roca J. Assessment of Hospital Avoidance in a Real-World Setting: a Prospective Cohort Study, Submitted), carried out during the same period, clearly showed higher performance and reduction of both hospital and community-based direct costs, compared to conventional hospitalization.

The Catalan healthcare system (7,7 million citizens), with one-single public payer and high heterogeneity of providers, is organized in three layers being the seven health regions at the top level. Each region includes several geographical areas called health districts covering specialized, intermediate, and primary care needs of the population. It is of note that intermediate care plays a key role facilitating vertical integration [31–34]. The third level, community-based teams, corresponds to clusters of primary care centres within each healthcare district. Catalonia has a total of 369 primary care units covering approximately 20,000 citizens, on average, each of them.

Regional deployment of the Chronic Care model [35] and integration of health and social services has been promoted under the umbrella of the five-yearly regional health plans. Key goals in terms of deployment of the integrated care were established during the 2011–2015 Plan and consolidation of the program was done during the 2016–2020 period [36, 37].

### Study groups and design for HaH-HA assessment

The current prospective cohort study performs a real-world data analysis with a Triple Aim approach of the first episode of a subset of non-surgical patients consecutively admitted to HaH-HA, directly from the Emergency Room Department (ER) over one year, from 31^st^ October 2017 to 1^st^ November 2018. Patients undergoing HaH-ED were not considered in the current study to have a more homogeneous intervention group. Inclusion/exclusion criteria, as well as characteristics of the intervention (HaH-HA) have been extensively reported in [28].

A control group (Usual Care, UC), under conventional hospitalization for their first acute episode during the study period, was generated with patients admitted through the ER directly to a general ward, excluding surgical wards and intensive care units, attempting to mimic patients’ clinical profiles between HaH-HA and UC. Comparability between HaH-HA and UC groups was improved with a 1-to-1 Propensity Score Matching (PSM) [38, 39] and Genetic Matching [40] taking simultaneously into account two sets of matching variables to ensure patients’ comparability before admission and during the acute episode.

The first set of matching variables were: i) age; ii) gender; iii) number of admissions during the previous year; iv) patient’s healthcare costs across the health system in the previous year; and v) patient’s population-based risk in terms of Adjusted Morbidity Groups (GMA) [41] scoring. Two additional matching variables, characterizing the acute episode, were included to enhance comparability of the two groups; that is: i) main diagnosis at discharge using the ICD10-CM categories, and ii) case mix index (CMI)[42]. In this regard, GMA is an aggregative index which indicate the burden of an individual’s morbid conditions through a disease-specific weighting deduced from statistical analysis based on mortality and the utilisation of health services; whereas CMI reflects both severity/complexity of main diagnosis, as well as the complications occurring during the admission.

The matching parameters were tuned according to overall performance on covariate balancing, assessed by the Mahalanobis distance [43], Rubin’s B (the absolute standardized difference of the means of the linear index of the propensity score in the HaH-HA and UC groups) and Rubin’s R (the ratio of HaH-HA to UC variances of the propensity score index) metrics [44]. Quality of comparability between HaH-HA and UC after PSM was considered acceptable if Rubin’s B was less than 0.25 and Rubin’s R was between 0.5 and 2**.**

### Triple aim assessment and MCDA of HaH-HA

As indicated above, HaH-HA was assessed with a Triple Aim approach and the outcomes were elaborated using MCDA. Briefly, we aimed to evaluate outcomes that go beyond traditional health variables and include patient reported outcomes and their broader sense of wellbeing, experience with care, and costs from a hospital perspective. For the MCDA, we used the following eight outcomes: i) enjoyment of life [45]; ii) resilience [46]; iii) physical functioning [47]; iv) continuity of care [48]; v) psychological wellbeing [49];(vi) social relationships & participation [50]; (vii) person-centeredness [51]; and, (viii) costs. The questionnaires for all items were administered by a nurse, at 30 days after discharge, in the patients’ home. Moreover, two of these questionnaires, explicitly: i) continuity of care; and ii) person-centeredness were administered again at 90 days after discharge.

Additionally, Discrete Choice Experiments (DCE) [52] were used to obtain weights for the following set of outcomes. They were elicited in an online weight elicitation study among five stakeholder groups: i) patients; ii) informal caregivers; iii) professional care providers; iv) payers; and v) policy makers (we pooled responses for payers and policy makers).

### Statistical analysis

For every key outcome, MCDA inputs for the performance scores were calculated using the following linear mixed regression models applied to the matched patient groups, missing data was not considered to build the performance regression models:

Where only T0 (30 days after discharge) available,

$$Y=constant + \beta 1^ * intervention$$

where variable time is available,

$$Y=constant+\beta 1^*time+\beta 2^*intervention+\beta 3^*time^*intervention$$

The resulting performance regression models were used to calculate corresponding predicted values. In each group, HaH-HA and UC, the mean score of the predicted values was standardized relatively to the mean score of the predicted values in both groups. The corresponding formulas are:

$${\mathrm{S}}_{\mathrm{ho}}=\frac{{\mathrm{x}}_{\mathrm{ho}}}{{\left({\mathrm{x}}_{\mathrm{ho}}^{2}+{\mathrm{x}}_{\mathrm{uo}}^{2}\right)}^{1/2}} , {\mathrm{ S}}_{\mathrm{uo}}=\frac{{\mathrm{x}}_{\mathrm{uo}}}{{\left({\mathrm{x}}_{\mathrm{ho}}^{2}+{\mathrm{x}}_{\mathrm{uo}}^{2}\right)}^{1/2}}$$

$$\mathrm x=\mathrm{performance}\;\mathrm{score}\;\mathrm{in}\;\mathrm{terms}\;\mathrm{of}\;\mathrm{mean}\;\mathrm{predicted}\;\mathrm{values}$$

$$\mathrm h=\mathrm{Hospital}\;\mathrm{at}\;\mathrm{Home}\;\mathrm{group}$$

$$\mathrm u=\mathrm{Usual}\;\mathrm{Care}\;\mathrm{group}$$

$$\mathrm o=\mathrm{performance}\;\mathrm{outcome}$$

For each stakeholder group, the mean predicted outcome values were weighted and subsequently summed to obtain a single overall value score for the HaH-HA group and a single overall value score for the UC group.

Finally, a Monte Carlo simulation addressed parameter uncertainty in both performance outcomes and weights. The required number of bootstrap replications (1,000 iterations) was determined checking stability of the results (95% uncertainty interval around the mean overall value scores).

### Population-health assessment using a Triple Aim approach

The population-health impact of the regional deployment of integrated care over the period 2011–2017 was assessed using registry data [20, 21]. The Catalan Health Surveillance System (CHSS) [20] was the main data source for analysis of the evolution of traditional health outcomes and costs on a yearly basis for the period 2011–2017; whereas the potential for a Triple Aim evaluation with a population-health approach; that is, including patient reported outcomes (PROMs) and patient reported experience (PREMs) was explored using the ESCA survey *(Enquesta de Salut de Catalunya)* [21]. This survey periodically assesses health status, behavioural changes in use of healthcare resources and perception of the healthcare system in Catalonia. The survey is done, on a yearly basis, on a randomly selected sample (*n* = six thousand citizens) of the Catalan population taking as a basis the seven healthcare regions.

The CHSS includes updated registries of the region of Catalonia from primary care, hospital-related events (hospitalizations, emergency room consultations and specialized outpatient visits), pharmacy, mental health, socio-sanitary services and other items (home-based respiratory therapies, dialysis, outpatient rehabilitation and non-urgent healthcare transportation) since 2011 [20]. It allows analyses on use of healthcare resources, pharmacy consumption, prevalence of key disorders and population-based health risk assessment. Data analysis can be done considering different levels of aggregation: Primary Care Units, Primary Care Areas, Health Districts, Health Regions or the entire Catalonia.

In the current study, the CHSS analysis of health outcomes was constrained to the temporal evolution of three selected KPI of quality of management of chronic patients: i) unplanned hospitalizations in emergency rooms of acute hospitals; ii) potentially avoidable hospitalizations; and iii) hospitalizations due to chronic conditions in acute hospitals. The analysis assumes that lower values for the 3 KPI reflect better health outcomes. In the study, we compared three different geographical areas: i) the health district of Barcelona-Esquerra (AISBE, 520 thousand citizens) wherein HCB is the reference hospital and pioneered in the deployment of integrated care services; ii) the entire city of Barcelona (1,7 million citizens) and iii) the entire Catalonia (7,7 million citizens). Rates of the 3 KPIs were adjusted by the corresponding KPI rate for Catalonia in each year, and a 95% confidence interval was fixed based on the Byar's approximation of the exact Poisson distribution which is extremely accurate even with small numbers [53].

### Statistics

Categorical variables were summarized as absolute values and frequencies, whereas continuous variables were represented by the mean and the standard deviation or the median and interquartile range, as appropriate. Unpaired Student T tests, Mann–Whitney and Chi-squared test comparing HaH-HA with UC were used to assess changes in the outcomes. Data analyses were conducted using R [54], version 3.6.1 The threshold for significance was set at 0.05.

## Results

### Triple Aim assessment and MCDA of HaH-HA

**Fig. 1:**
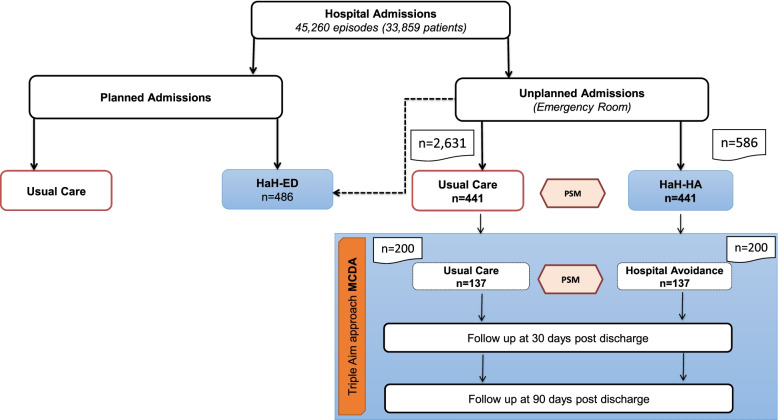
Distribution of hospital admissions during the study period. Five**-**hundred eighty-six first episodes of HaH admissions, directly from the Emergency Room (HaH-HA), were registered during the study period. A sample of 2.631 conventional hospitalizations was used to generate a usual care (UC) group, as described in the text. The entire intervention group, after propensity score matching (PSM), consisted of 441 HaH-HA patients that were compared with the corresponding matched controls (UC), as reported in (Carme H, Carme H, Erik B, Nuria S, Ruben G, Asenjo M, David N, Enric C, Fernandez J, Isaac C, Roca J. Assessment of Hospital Avoidance in a Real-World Setting: a Prospective Cohort Study, Submitted) During the study period, two-hundred consecutive HaH-HA patients were assessed with a Triple Aim approach to perform Multiple Criteria Decision Analysis (MCDA) that was finally done in 137 HaH-HA patients after PSM with a UC group. Comparisons between the entire HaH-HA population (*n* = 586), the CCA study (*n* = 441) and the current study (*n* = 137) are reported in Tables 1S-3S

Figure 1 displays the distribution of hospital admissions to conventional hospitalization and to the HaH program: HaH-ED and HaH-HA during the study period. A total of 200 consecutive HaH-HA patients, and their corresponding controls, were studied as candidates for Triple Aim assessment. Application of PSM techniques described above reduced the study groups to 137 HaH-Ha cases, and the corresponding controls.

Table 1 displays the baseline characteristics of the two groups before admission and the clinical outcomes during the acute episode until 30 days after discharge. The comparability analysis between HaH-HA and UC shows a highly acceptable matching. It is of note that the two groups showed no differences in mortality. However, patients under HaH-HA presented significantly lower rates of emergency room visits (*p* =  < 0.001) and hospital admissions, unplanned (*p* = 0.012) and planned (*p* = 0.031), during the 30-day period after discharge than the UC group.

**Table 1:** Characteristics of the study groups after propensity score matching before admission and clinical outcomes after discharge

	**HaH-HA_MCDA**	**UC_MCDA**	***P- value***
	**(*n* = 137)**	**(*n* = 137)**	
**BASELINE CHARACTERISTICS**			
**Socio-demographics**			
Age (years), mean (SD)*	72.42 (13.92)	72.79 (15.37)	.836
Gender (male), n (%)*	80 (58.39)	79 (57.66)	.902
Gender (female), n (%)*	57 (41.61)	58 (42.34)	.902
**Use of healthcare resources**			
***Hospital resources in previous 12 months***			
Rate of all-cause emergency room visit, mean (SD)	1.85 (1.19)	1.76 (1.23)	.231
Rate of all-cause Hospital admissions, mean (SD)*	1.45 (0.9)	2.02 (1.54)	.171
Rate of planned admissions, mean (SD)	1.32 (0.67)	1.67 (0.91)	.152
Last visit (days) to outpatient clinic before admission, mean (SD)	78.36 (86.09)	78.07 (81.86)	.981
Last hospitalisation (days) before admission, mean (SD)	202.12 (108.21)	222.25 (110.74)	.413
Length of stay in days (total days per year), mean (total)	11.45 (458)	14.75 (590)	.408
Intensive care unit stays, n (%)	7 (12.10)	4 (5.70)	.405
Outpatient visits, mean (SD)	6.14 (7.19)	5.64 (5.55)	.604
***Hospital resources in previous 7 days***			
Outpatient visits, mean (SD)	1.20 (0.63)	1.25 (0.62)	.405
***Healthcare costs across tiers in previous year***			
€ per year, mean (SD)*	7,023.81 (9,478.93)	8,138.43 (8,083.83)	.854
**Multimorbidity and severity**			
GMA scoring, mean (SD)*	27.93 (14.72)	28.8 (18.74)	.872
**CLINICAL OUTCOMES AT DISCHARGE**			
**Total length of stay (days), mean (SD)**	8.20 (4.70)	7.74 (5.96)	.479
**Case Mix Index**	0.72	0.75	.792
**Mortality during episode, n (%)**	0 (0)	0 (0)	1
**Use of resources during Hospital Avoidance**			
All-cause Emergency Room visits, n (%)	2 (1.46)	N/A	NA
All-cause In-Hospital re-admissions, n (%)	4 (2.92)	N/A	NA
**Outcomes at 30 days after discharge**			
All-cause Emergency Room visits, n (%)	5 (3.65)	20 (14.6)	< .001
Unplanned Hospital admissions, n (%)	5 (3.65)	15 (10.95)	.012
Planned admissions, n (%)	2 (1.46)	9 (6.57)	.031
Mortality, n(%)	1 (0.73)	1 (0.73)	1
**MATCHING ASSESSMENT METRICS**			
**Rubin's B**	**0.050**		
**Rubin's R**	**0.598**		

Legend. HaH-HA, Hospital al Home-Hospital Avoidance; UC, Usual Care; GMA, Adjusted Morbidity Groups scoring; N/A, not applicable

The raw data of the eight outcomes before the MCDA (Table 2) indicate that HaH-HA group showed slightly better physical functioning (*p* = 0.019), and higher scores for both patient-reported experience measures: continuity of care (*p* < 0.001) and person-centeredness (*p* < 0.001) than the UC group.

**Table 2:** Raw data of the eight outcomes before the MCDA

**Criteria/Outcomes**	**Questionnaire**		**Score: Mean (SD)**		***P*-value**
		**Range**	**HaH-HA**	**UC**	
			***n* = 137**	***n* = 137**	
Enjoyment of life	ICECAP-O	1–4	2.50 (0.89)	2.48 (0.87)	.496
Resilience	BRS	6–30	20.52 (5.48)	19.72 (5.99)	.278
Physical functioning	SF-36	0–100	55.27 (35.43)	45.45 (31.87)	.019
Continuity of care	NCQ	0–5	3.82 (1.20)	3.30 (1.15)	< .001
Psychological well-being	MHI-5	0–100	69.32 (23.52)	68.07 (22.11)	.623
Social participation	IPA	0–24	7.13 (3.95)	7.00 (3.77)	.768
Person-centeredness	P3CEQ	0–18	15.94 (2.95)	14.62 (3.80)	.001
Health care costs	€		1,126.76 (226.17)	2,346.33 (519.14)	< .001

Results expressed as mean (standard deviation). Questionnaires used for the MCDA (Multiple Criteria Decision Analysis) and CCA (Cost Consequence Analysis) are indicated in the Methods section. HaH-HA, (Hospital at Home-Hospital Avoidance); UC, Usual Care

Two outcomes reflecting patients’ experience with care: i) continuity of care; and ii) person-centeredness (Table 2, 30 days after discharge) were also assessed at 90 days after discharge without showing significant changes: mean change (95%CI), 0.022 (-0.25–0.29) and -0.3 (-1.16–0.51), respectively.

The direct HaH-HA costs per patient during the episode was, on average 1,127€; whereas the direct UC costs per patient was slightly more than double, mean 2,346€. It is of note that the average cost savings per patient in HaH-HA (1,220€) were mostly generated (95%) by a reduction in staff (54%), testing (17%), catering (12%) and infrastructure (12%) costs (Table 3S). Likewise, healthcare expenses across the health system during the 30-days transitional care period after discharge; that is, including use of healthcare resources at community level, were 62% lower in HaH-HA than in UC (*p* < 0.001).

**Fig. 2:**
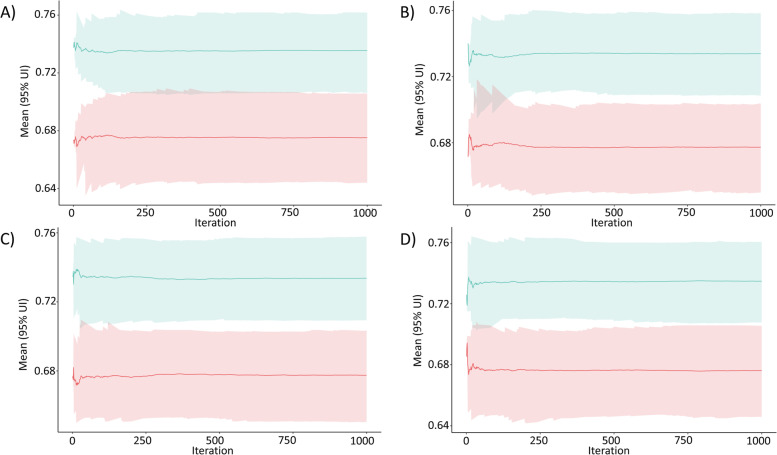
Sensitivity analysis of MCDA with DCE weights based on a Bootstrap analysis (1,000 iterations) of the MCDA overall score between hospital avoidance (HaH-HA) (green) and usual care (UC) (red) groups. In each iteration, the values of the eight outcomes considered in the MCDA were weighted according to the DCE results and subsequently summed to obtain a single overall value score. The panels show the mean overall value score across all bootstrap iterations (set to 1000) and their 95% Uncertainty Intervals (UI) for HaH-HA and UC and for each stakeholder group: **A**) Patients; **B**) Carers; **C**) Professionals; **D**) Payers + Policy Makers. The four panels show no overlap between intervention and control groups along the bootstrap replications

The main results of the MCDA of HaH-HA are depicted in Table 3. The table indicates standardized values of each of the outcomes indicating (PROMs) and patient experience with care (PREMs), as well as health care costs, weighted by the opinions of the stakeholder groups, i.e.: patients, carers, health professionals and policy makers/payers. It is of note that from all stakeholder perspectives, HaH-HA consistently showed higher overall value scores than UC (Table 4S). In addition, the results of the Monte Carlo simulation disclosed that the weighted aggregation of the performance scores is significantly greater in HaH-HA group, showing no overlap of the corresponding 95%CI. This finding is also observable assessing the intervention using the weighting preferences from every stakeholder, showing no overlap of the corresponding 95%CI (Fig. 2).

**Table 3:** Overall scores of the multiple criteria decision analysis (MCDA)

		**Case Mix MCDA Sub-Group**			
		**(*n* = 137)**			
**Criteria/Outcomes**	**Relative Weights**	**Standardized performance**		**Weighted aggregation**	
		**HaH-HA**	**UC**	**HaH-HA**	**UC**
Enjoyment of life	0.22	0.71	0.71	0.15	0.15
Resilience	0.13	0.72	0.69	0.09	0.09
Physical functioning	0.12	0.77	0.64	0.09	0.07
Continuity of care	0.15	0.75	0.66	0.11	0.10
Psychological well-being	0.14	0.71	0.70	0.10	0.10
Social participation	0.11	0.72	0.70	0.08	0.08
Person-centeredness	0.09	0.74	0.67	0.07	0.06
Health care costs	0.05	0.87	0.49	0.04	0.02
**Overall value score**				**0.73**	**0.67**
**(95% CI)**				**(0.71–0.76)**	**(0.65–0.70)**
**Percentage HaH-HA > UC**				**98.4**	

Criteria/Outcomes: 8 outcomes categories assessed in the MCDA at 30 days after discharge; Relative weights: pooled weights of all five stakeholder groups; Standardized performance: Overall scoring for each outcome, HaH-HA: Hospital at Home-Hospital Avoidance, UC: Usual Care; Weighted aggregation: Standardized performance times corresponding relative weight. HaH-HA, Hospital at Home-Hospital Avoidance; UC, Usual Care; Overall value score: mean overall scores for HaH-HA and UC; 95% Uncertainty Interval; Percentage HaH-HA > UC, percentage of iterations in the Monte Carlo simulation showing higher overall value scores in HaH-HA than UC.

### Population-health assessment using a Triple Aim approach

**Fig. 3:**
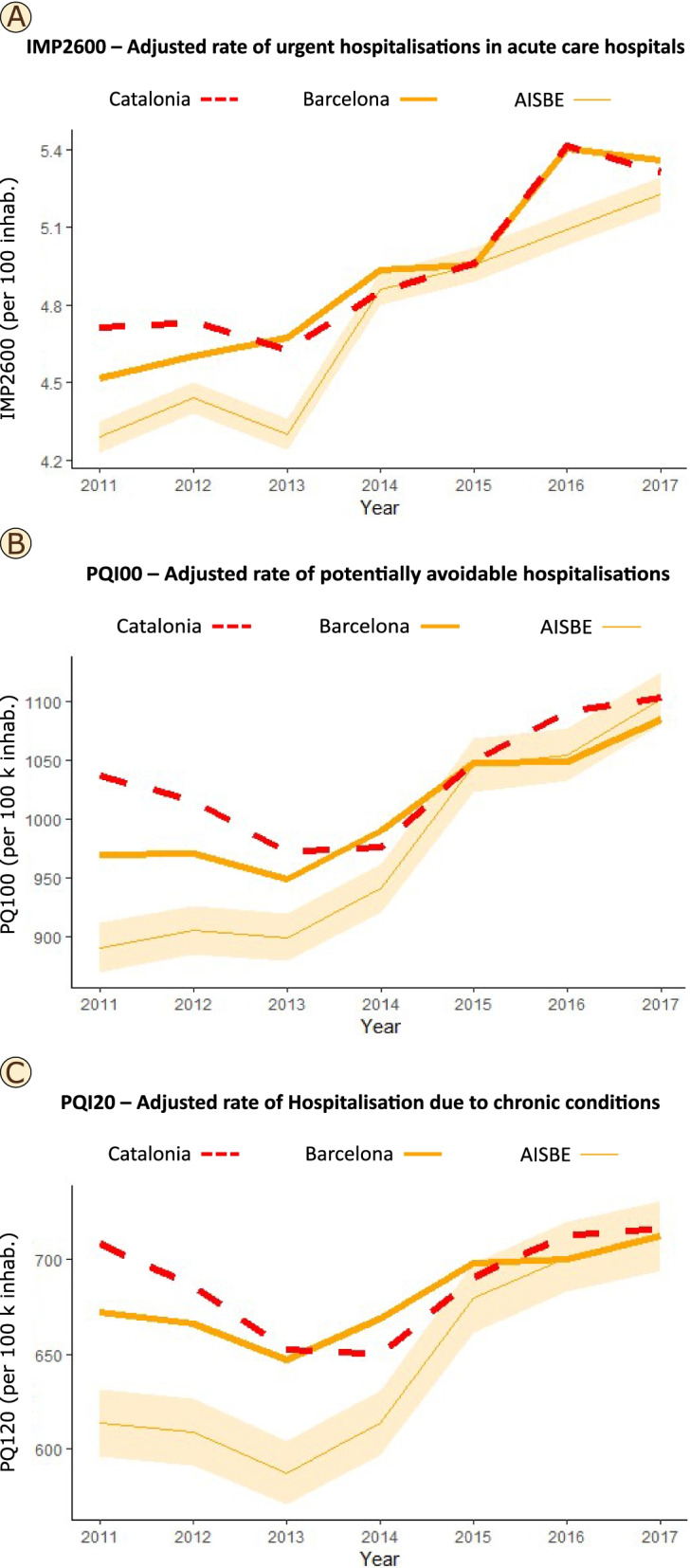
Quality of chronic care in the study areas

Figure 3 displays the yearly evolution, from 2011–2017, of the three selected KPI reflecting quality of chronic care in the study areas: i) health district of Barcelona-Esquerra; ii) city of Barcelona; and iii) Catalonia. From top to bottom, the panels depict adjusted rates per 10 thousand citizens for: i) unplanned ER visits in acute hospitals, ii) potentially avoidable hospitalizations, and iii) hospitalizations due to chronic conditions in acute hospitals. Over the period 2011–2014, both avoidable hospitalizations and hospitalizations of chronic patients showed a reduction in both the city of Barcelona and the entire Catalonia; whereas AISBE clearly presented a plateau with lower adjusted rates for all KPI than Barcelona and Catalonia. Interestingly, all KPIs in the three geographical areas presented a sustained increase (worsening) after 2013, reaching convergence at the end of the study period. The CHSS allowed such analysis, with different levels of data aggregation, for a large amount of health outcomes and for costs.

The feasibility analysis of the ESCA survey to assess PROMs and PREMs by geographical areas identified some limitations. The ESCA outcomes could partly be mapped to the MCDA variables displayed in Tables 2 and 3. Briefly, four domains: i) psychological well-being; ii) social relationships and participation; iii) resilience; and iv) enjoyment of life were assessed with well-identified measurement tools and showed equivalences with specific MCDA outcomes. Other three domains did not have a standardized measurement tool but showed some equivalences with specific MCDA outcomes, namely: i) physical functioning; ii) activation and engagement; and iii) person centeredness. Only one domain, continuity of care, could not be mapped into the ESCA survey. Moreover, the analysis of the yearly evolution, by geographical areas, of the ESCA outcomes could not be done because of two main technical issues. Firstly, changes introduced in ESCA during 2016–2017 did not allow comparability during the entire study period. A second limitation was that the characteristics of the sampling: size and distribution of data did not allow appropriate comparisons among the geographical areas considered in the current research. Up to 4000 surveys were done in the city of Barcelona with unknown distribution by health districts and approximately 400 surveys corresponded to each of the other six health regions.

## Discussion

### Hospital avoidance: main findings and context of the analysis

The current report provides a value-based assessment of Hospital at Home-hospital avoidance taking into consideration health and well-being, experience with care and healthcare costs, considering the acute episode and the 30-day period post-discharge. The MCDA clearly showed that HaH-HA was superior to UC, for those candidates meeting the inclusion/exclusion criteria. The research, taking HaH-HA as use case, clearly shows feasibility and applicability of the Triple Aim approach for comprehensive assessment of specific integrated care services, as proposed in [19]. The results are along with those seen in the retrospective CCA carried out with the entire population of patients included in HaH-HA during the study period (Carme H, Carme H, Erik B, Nuria S, Ruben G, Asenjo M, David N, Enric C, Fernandez J, Isaac C, Roca J. Assessment of Hospital Avoidance in a Real-World Setting: a Prospective Cohort Study, Submitted), which strengthens the messages of the current research and reinforces the interest for a comprehensive health delivery assessment (Tables 1S, 2S and 3S). It is of note that cost-savings associated to HaH-HA are mostly attributable to organizational changes and digital support of the service rather than to specificities of the patients’ traits. Accordingly, our results can be reasonably generalized for this type of intervention provided that the general characteristics of the HaH-HA service are maintained [55].

### Triple aim assessment of specific integrated care services

The use of MCDA allowed measured outcomes to be balanced by the opinions of relevant stakeholders which shows high potential to facilitate decision making at policy level. We believe that the HaH-HA study provides a methodological approach that can be easily generalizable for assessment of other ICS involving complex interventions.

It is of note that our research aimed to explore applicability of the Triple Aim approach for assessment of ICS in real world settings. One key limiting finding was that the administration of the questionnaires assessing the seven qualitative variables included in the MCDA required approximately 45 min per patient which precludes use in routine testing. Pilot testing carried out before the initiation of the study protocol indicated that administration of questionnaires earlier during admission might not provide robust results because of limitations for data capture due to patients’ clinical conditions. Moreover, the lack of significant changes between 30-days and 90-days after discharge might indicate the need for improving discriminative power of administered questionnaires.

One lesson learnt is that capturing relevant information from patients in real-world scenarios requires refinement of the measurement tools including questionnaires and characteristics of the digital support. We could hypothesize that comprehensive outcomes’ assessment using properly validated visual-analogic scales and/or artificial intelligence-powered brief questionnaires [56] could be alternative approaches to be explored for a desirable adoption of Triple Aim assessment in routine healthcare delivery assessment. The use of user-friendly digital tools conceived to support collaborative work and adaptive case management, as described in [57], should greatly facilitate capturing patients’ information. Such digital tools should also allow a fluent capture of professional inputs allowing Quadruple Aim [16] assessment of healthcare services.

### Lessons learnt from population-health study

It is widely accepted that generation of evidence on value-based integrated care at health system level is still an unmet need. The current study indicates that the characteristics of the CHSS fulfill the requirements of health policy makers at regional level and constitute an appropriate tool for assessment of traditional health outcomes, as well as analysis of associated direct costs. In contrast, the ESCA survey shows clear limitations for a Triple Aim assessment that could be easily by overcome by refining the digital support by capturing citizens’ answers with visual-analogic scales and modifying the sampling characteristics aiming at enhancing robustness of the analysis of specific geographical areas.

The results displayed in Fig**. ****3** reflect highly complex phenomena and may require deeper analysis for a proper understanding of the information. A possible interpretation of the evolution of the selected KPIs indicating quality of chronic care could be as follows. Convergence of selected KPIs between AISBE (early adopter of integrated care services since 2006), the city of Barcelona and the region of Catalonia might be explained by preparation and active deployment of the 2011–2015 Catalan Health Plan heavily promoting integrated care and digitalization of healthcare. However, worse health outcomes in the three areas after 2013 might likely reflect the negative effects of a marked (-10%) reduction of financial healthcare resources during the period 2011–2018, due to the 2008 economic crisis.

### Future perspectives

In our setting, the 2011–2015 Health Plan for Catalonia (Spain) stimulated a substantial expansion of HaH with various approaches and overall positive results. It is of note that the successful regional deployment of HaH during the period 2015–2019 is currently being analysed as an original Good Practice (oGP) within the Joint Action on implementation of digitally enabled integrated person-centred care (JADECARE, 2020–2023). We believe that proposals generated by the current research should trigger improvements in the assessment integrated care services facilitating comparability and transferability of Good Practices across sites.

## Conclusions

The current study performed a comprehensive assessment of HaH-HA in a real-life setting using the novel methodological approach of MCDA and produced evidence on health value generation of the intervention. The research identifies key actionable factors for Triple Aim assessment of the impact of integrated care interventions at health system level.

## Supplementary Information

**Additional file 1.**

## Data Availability

The datasets generated and/or analyzed during the current study are not publicly available due to administrative reasons but are available from the corresponding author on reasonable request.

## Abbreviations

HaH: Hospital at home
HaH-HA: Hospital avoidance
UC: Conventional hospitalization
MCDA: Multiple criteria decision analysis
ER: Emergency room department
KPI: Key performance indicators
ICS: Integrated care services
HaH-ED: Early discharge
CCA: Cost-consequence analysis
PSM: Propensity score matching
DCE: Discrete choice experiments
CHSS: Catalan health surveillance system
PROM: Patient reported outcome
PREM: Patient reported experience
ESCA: Catalan health survey
AISBE: The health district of Barcelona-Esquerra
GMA: Adjusted Morbidity Groups scoring
oGP: Original Good Practice
