# Supplementary material for: Prospective cohort study for assessment of integrated care with a triple aim approach: hospital at home as use case
Source: BMC Health Serv Res. 2022 Sep 7;22:1133. doi: 10.1186/s12913-022-08496-z (PMC9454140; doi:10.1186/s12913-022-08496-z)
Supplement: Supplementary file 1 — Additional file 1. [file 12913_2022_8496_MOESM1_ESM.docx]

**Prospective Cohort Study for Assessment of Integrated Care with a Triple Aim approach: Hospital at Home as use case**

*(on-line supplementary material)*

*Herranz C. et al*

**COMPARISONS AMONG STUDY GROUPS (TABLES 1S-3S) AND MCDA (TABLE 4S)**

**Tables 1S-3S** depict comparisons between intervention and control groups across the flowchart displayed in **Figure 1**, that is: i) the entire population of patients included in the Hospital at Home program directly from the Emergency Room, Hospital Avoidance (n=586), ii) the selected subset after propensity score matching (n=441) reported in the Cost-Consequence Analysis (CCA) (ref); and, iii) the Triple Aim analysis carried out in the current study (n=137). The ultimate aim is to reflect representativeness of the conclusions obtained from the current Triple Aim assessment of HaH-HA.

**Table 1S** displays baseline data of the entire HaH-HA population (n=586), the HaH-HA group (n= 441) selected after Propensity Score Matching (PSM) and the corresponding usual care (UC) group. Moreover, the table also displays baseline characteristics of the MCDA subsets after PSM (n=137): HaH-HA_MCDA and the corresponding usual care group (UC-MCDA).

**Table 2S** provides data during the acute episode and during the 30-day period after discharge for the same datasets described in **Table 1S**.

**Table 3S** depicts information on operational costs of the service during the acute episode, as well as the community-based expenditure during the 30-day period after discharge following the same organization of the information indicated for **Tables 1S** and **2S**.

**Table 4S** provides the results of the sensitivity analysis **of** MCDA from all stakeholders’ perspectives calculated using Discrete Choice Experiments (DCE) weights.

**TABLE 1S. Characteristics of the study population (n=586) and study groups (CCA, n=441 and MCDA n=137) after propensity score matching, before admission**

***Legend.*** *CCA, Cost Consequence Analysis; Study population: all patients included in Hospital at Home-Hospital Avoidance (HaH-HA); HaH-HA, corresponds to the study population after propensity score matching (PSM); UC, Usual Care, after PSM; MCDA, multiple criteria decision analysis; HaH-HA_MCDA, intervention subset used for MCDA, after PSM; UC_MCDA, usual care subset used for MCDA, after PSM; GMA, Adjusted Morbidity Groups scoring; * Matching variables.*

**TABLE 2S. Characteristics of the acute episode and main outcomes for the entire population of HaH-HA and for the two study groups: CCA (n=441) and MCDA (n=137)**

***Legend.*** *CCA, Cost Consequence Analysis; Study population: all patients included in Hospital at Home-Hospital Avoidance (HaH-HA); HaH-HA, corresponds to the study population after propensity score matching (PSM); UC, Usual Care, after PSM; MCDA, multiple criteria decision analysis; HaH-HA_MCDA, intervention subset used for MCDA, after PSM; UC_MCDA, usual care subset used for MCDA, after PSM.*

**Table 3S. Operational Cost (in €) during the acute episode and expenses during 30-days after discharge for the different study groups**

***Legend.*** *Study population: all patients included in Hospital at Home-Hospital Avoidance (HaH-HA); HaH-Ha, corresponds to the study population after propensity score matching (PSM); UC, Usual Care, after PSM; HaH-HA_MCDA, intervention subset used for multiple criteria decision analysis (MCDA), after PSM; UC_MCDA, usual care subset used for MCDA, after PSM.*

| **Criteria/Outcomes** | **Standardized performance** | | **Patients** | | | **Informal caregivers** | | | **Professional care providers** | | | **Payers & Policy makers** | | |
| --- | --- | --- | --- | --- | --- | --- | --- | --- | --- | --- | --- | --- | --- | --- |
|  |  |  | **Weights** | **Weighted aggregation** | | **Weights** | **Weighted aggregation** | | **Weights** | **Weighted aggregation** | | **Weights** | **Weighted aggregation** | |
|  | **HaH-HA** | **UC** |  | **HaH-HA** | **UC** |  | **HaH-HA** | **UC** |  | **HaH-HA** | **UC** |  | **HaH-HA** | **UC** |
| Enjoyment of life | 0,71 | 0,71 | 0,21 | 0,15 | 0,15 | 0,23 | 0,16 | 0,16 | 0,21 | 0,15 | 0,15 | 0,20 | 0,14 | 0,14 |
| Resilience | 0,72 | 0,69 | 0,14 | 0,10 | 0,10 | 0,13 | 0,09 | 0,09 | 0,12 | 0,09 | 0,08 | 0,13 | 0,09 | 0,09 |
| Physical functioning | 0,77 | 0,64 | 0,15 | 0,11 | 0,09 | 0,09 | 0,07 | 0,06 | 0,10 | 0,08 | 0,07 | 0,12 | 0,09 | 0,08 |
| Continuity of care | 0,75 | 0,66 | 0,14 | 0,11 | 0,09 | 0,16 | 0,12 | 0,10 | 0,15 | 0,11 | 0,10 | 0,14 | 0,10 | 0,09 |
| Psychological well-being | 0,71 | 0,70 | 0,13 | 0,09 | 0,09 | 0,15 | 0,10 | 0,10 | 0,14 | 0,10 | 0,10 | 0,15 | 0,10 | 0,10 |
| Social participation | 0,72 | 0,70 | 0,10 | 0,07 | 0,07 | 0,11 | 0,08 | 0,07 | 0,11 | 0,08 | 0,08 | 0,11 | 0,08 | 0,08 |
| Person-centeredness | 0,74 | 0,67 | 0,08 | 0,06 | 0,05 | 0,09 | 0,07 | 0,06 | 0,11 | 0,08 | 0,07 | 0,09 | 0,07 | 0,06 |
| Health care costs | 0,87 | 0,49 | 0,04 | 0,03 | 0,02 | 0,04 | 0,04 | 0,02 | 0,04 | 0,04 | 0,02 | 0,07 | 0,06 | 0,03 |
| **Overall value score** | | |  | **0,74** | **0,68** |  | **0,73** | **0,67** |  | **0,73** | **0,68** |  | **0,74** | **0,67** |
| **(95% UI)** | | |  | **(0.710-0.761)** | **(0.645-0.703)** |  | **(0.713-0.757)** | **(0.650-0.700)** |  | **(0.715-0.755)** | **(0.654-0.698)** |  | **(0.711-0.756)** | **(0.651-0.702)** |
| **Percentage IC > UC** | | | **98,8** | | | **99,6** | | | **99,7** | | | **99,3** | | |

**Table 4S. Sensitivity analysis of MCDA from all stakeholders’ perspectives calculated using Discrete Choice Experiments (DCE) weights**

***Legend.*** *Criteria/Outcomes: 8 outcomes categories assessed in the MCDA at 30 days after discharge; Standardized performance: Overall scoring for each outcome for pooled data of all stakeholder groups. For each stakeholder group: Patients, Informal care givers, Professional care providers and Payers & Policy makers, the columns indicate the relative weights and weighted aggregation; that is standardized performance times corresponding relative weight. HaH-HA: Hospital at Home-Hospital Avoidance, UC: Usual Care; Overall value score: mean overall scores for HaH-HA and UC; 95% Uncertainty Interval; Percentage HaH-HA>UC, percentage of iterations in the Monte Carlo simulation showing higher overall value scores in HaH-HA than UC.*

*.*
